# Effect of Silica-Based Abrasive Ingredients on Fluoride Concentration in Toothpaste: An In Vitro and In Vivo Study

**DOI:** 10.3390/dj14090584

**Published:** 2026-09-10

**Authors:** Shuma Hamaguchi, Tatsuya Akitomo, Masashi Ogawa, Satoru Kusaka, Ryota Nomura

**Affiliations:** 1 Department of Pediatric Dentistry, Graduate School of Biomedical and Health Sciences, Hiroshima University, Hiroshima 734-8553, Japan; syuumai@hiroshima-u.ac.jp (S.H.); mo.caries0@gmail.com (M.O.); rnomura@hiroshima-u.ac.jp (R.N.); 2 Department of Pediatric Dentistry, School of Dentistry, Osaka Dental University, Osaka 573-1121, Japan; kusaka-s@cc.osaka-dent.ac.jp

**Keywords:** abrasive ingredients, fluoride, toothpaste

## Abstract

**Background/Objectives**: Toothpastes have recently been developed with purposes ranging from strengthening teeth and restoring periodontal tissue to removing stains and alleviating tooth sensitivity. Although the individual effects of each component are well understood, their interactions with each other remain unclear. **Methods**: The oral fluoride concentration following the use of dentifrices with different abrasive contents was evaluated in seven healthy adults. In addition, in in vitro apatite pellet experiments, fluoride concentration from the pellets was compared after the use of dentifrices with different abrasive contents. **Results**: A comparison of fluoride ion concentrations in the oral cavity after using five different types of toothpaste showed that toothpaste without abrasive ingredients retained the highest concentration at all time points from 5 min onward. When the concentration of abrasive ingredients was adjusted in the same toothpaste and compared, the toothpaste with no abrasive (0%) resulted in the highest fluoride concentration compared to all the other toothpastes (10%, 20%, 30%) at 10, 20 and 30 min. The concentration of fluoride released from apatite pellets was also highest in the toothpaste without abrasive ingredients. **Conclusions**: These results suggest that abrasive ingredients affect the concentration of fluoride in the oral cavity, and the effect may be particularly pronounced in hard tissues such as teeth.

## 1. Introduction

Dental caries is a common chronic infection caused by oral bacteria that seriously threatens oral and general health [1]. Although the incidence of dental caries has decreased significantly worldwide, many patients still suffer from dental caries [2,3]. Fluoride promotes remineralization of dental hard tissues and affects bacterial activity, and is therefore used clinically to prevent dental caries [4]. Regular toothbrushing is recommended to prevent decay and other oral diseases, and toothbrushing for 2 min twice daily with a fluoride toothpaste is generally recommended [5]. Although the maximum permissible fluoride concentration in toothpaste varies by age and country, it is reported that toothpaste containing 1000 to 1250 ppm fluoride is more effective than non-fluoride toothpaste [5].

Toothpaste contains various ingredients in addition to fluoride [6]. Extrinsic tooth discoloration is a common cosmetic problem, and toothpaste is recognized as an option for the removal and prevention of extrinsic staining while promoting a whiter tooth color [7,8]. Manufacturers enhance the whitening efficacy of toothpaste by incorporating specific abrasives and chemical agents [8]. However, consideration should be given to ensure that the additives do not compromise the fluoride’s effectiveness [6].

As the anticaries effect of fluoride is generally attributed to low but sustained fluoride concentrations in dental plaque and saliva, it is important for fluoride to remain in the oral cavity for a prolonged period [9]. Although considerable research has been conducted on additives that strengthen teeth when combined with fluoride, few studies have focused on the effect of abrasive ingredients on the persistence of fluoride concentrations [10,11]. In this study, we investigated the effect of the presence or absence of abrasives on the concentration of fluoride in the oral cavity after toothbrushing.

## 2. Materials and Methods

### 2.1. Research Involving Human Subjects

#### 2.1.1. Ethics Statement

This study was conducted in full adherence to the Declaration of Helsinki and approved by the Hiroshima University Epidemiological Research Ethics Committee (Approval number: E2024-0162). Prior to specimen collection, all subjects were informed of the study protocol and provided written informed consent.

#### 2.1.2. Subjects

Healthy adults in their 20s (four male and three female) participated in the study. They were instructed to restrict eating, drinking, and gargling for 2 h prior to the study. To minimize variability in salivary flow and other physiological conditions, all study sessions were conducted in the afternoon. Because the toothpastes were used in the oral cavity, complete blinding of each toothpaste was not feasible due to differences in taste. However, this study was conducted with the abrasive formulations blinded.

#### 2.1.3. Toothpastes

Five types of commercially available toothpaste containing 1450 ppm fluoride were used in the present study. Three of the toothpastes contained abrasive ingredients (toothpastes A–C), and two contained no abrasive ingredients (toothpastes D and E). The characteristics of the abrasive ingredients in each toothpaste are presented in Table 1.

**Figure 1:**
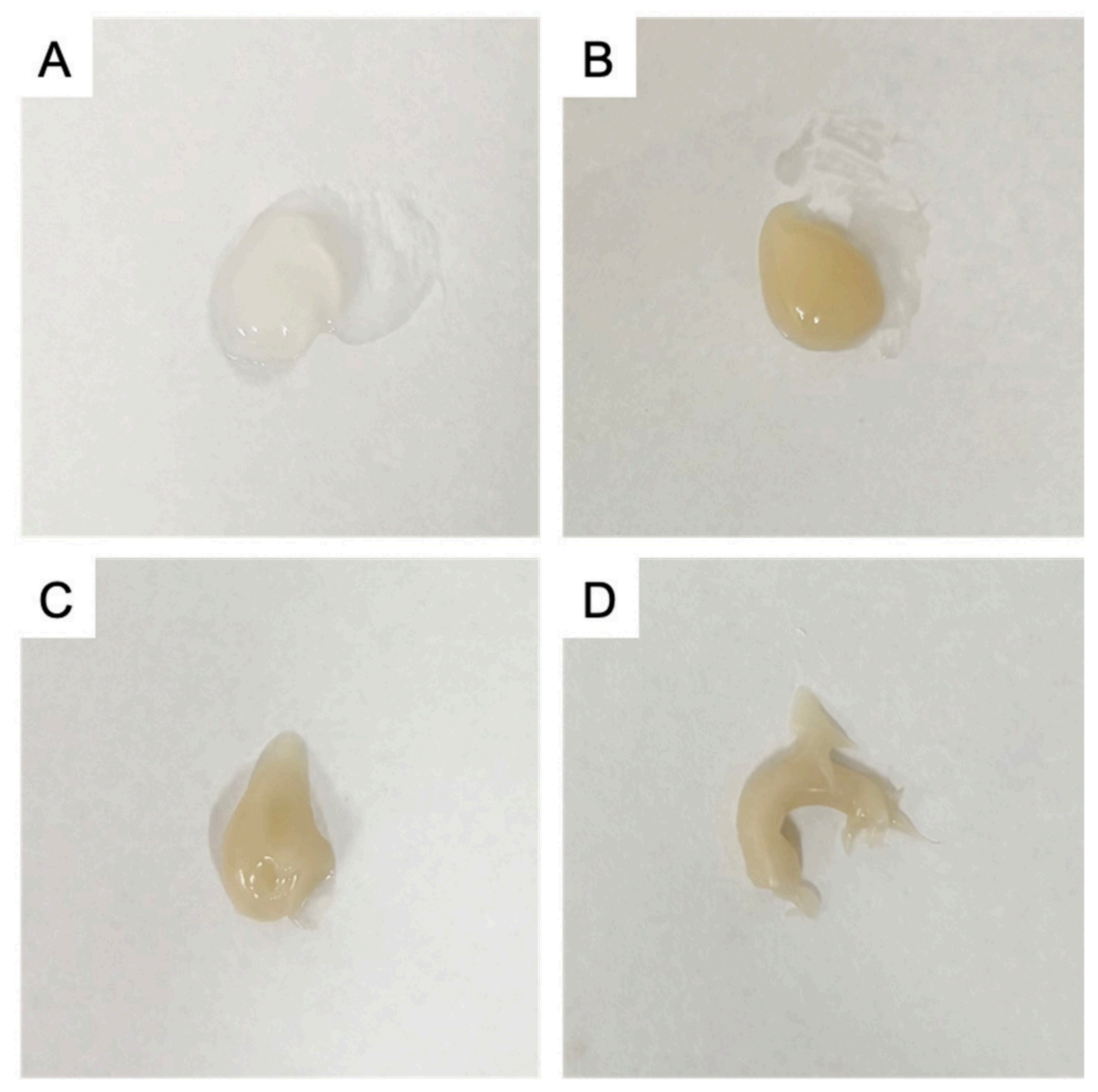
Images of toothpastes with different amounts of abrasive ingredients. (**A**) 0%, (**B**) 10%, (**C**) 20%, (**D**) 30%.

Samples of toothpaste D containing 1450 ppm fluoride were prepared with abrasive components adjusted to 10%, 20%, and 30%. Toothpaste D without abrasive components (0%) was used as a control. Images of each toothpaste are shown in Figure 1.

#### 2.1.4. Fluoride Concentration of Commercially Available Toothpastes

Using toothbrushes provided by Weltec (Osaka, Japan), participants brushed their teeth with 0.5 g of commercially available toothpaste for 2 min, and rinsed once with 10 mL of water for 5 s. They gargled again with 10 mL of water for 5 s (time point 0 min). They continued to rinse their mouth in the same way at 5, 10, 20, and 30 min after toothbrushing (time points 5 min, 10 min, 20 min, 30 min). Participants used five different toothpastes (A–E) on different days, and the solution they spat out after gargling was collected. Each solution was diluted with an equal amount of Total Ionic Strength Adjustment Buffer (HORIBA Ltd., Kyoto, Japan), and then the concentration of fluoride ions was measured using a Fluoride Ion Electrode (HORIBA Ltd., Kyoto, Japan). Measurements were performed after calibration using 100 mg/L and 1000 mg/L fluoride ion standard solutions. The limit of detection was 20 μg/L. Measurements were conducted after confirming the stability of the electrode response through repeated measurements.

#### 2.1.5. Fluoride Concentration of Toothpaste with or Without Abrasive Components

Because Toothpaste D used in Section 2.1.4 was manufactured by Weltec, formulations containing 10%, 20%, and 30% abrasive were prepared by the manufacturer. All formulations had the same base composition except for the abrasive concentration and were used as the toothpaste in this study. Using toothbrushes provided by Weltec, participants brushed their teeth with 0.5 g of toothpaste for 2 min, and rinsed once with 10 mL of water for 5 s. The subsequent steps were the same as in Section 2.1.4.

### 2.2. Research Involving Hydroxyapatite

#### 2.2.1. Materials

Apatite pellets, which are hydroxyapatite molded into a square shape (10 × 10 × 2 mm) and sintered, were purchased from HOYA Technosurgical Co. Ltd. (Tokyo, Japan).

#### 2.2.2. Fluoride Concentration of Apatite Pellets After Toothbrushing

Portions (0.5 g) of toothpaste with different concentrations of abrasive components (as used in Section 2.1.5) were placed on a toothbrush (Oral Care, Tokyo, Japan) and brushed back and forth 10 times on each side of the apatite pellet with the contact pressure adjusted to 20 g. The pellet was transferred to 5 mL of water and the 30-s stirring process was repeated three times. The remaining solutions were designated as solution 1, solution 2, and solution 3. Each solution was diluted with an equal amount of Total Ionic Strength Adjustment Buffer (HORIBA Ltd.), and the concentration of fluoride ions was measured using a Fluoride Ion Electrode (HORIBA Ltd.). Data were calculated as follows: (fluoride ion concentration of each solution−fluoride ion concentration of each solution when apatite pellets brushed without toothpaste were added).

### 2.3. Statistical Analysis

Statistical analyses were conducted using GraphPad Prism 9 (GraphPad Software Inc., La Jolla, CA, USA). Assays involving human subjects were carried out seven times, and assays using apatite pellets were carried out three times. Results were presented as means ± standard deviation. Statistical significance was determined by repeated-measures two-way analysis of variance (ANOVA), followed by Tukey’s multiple comparisons test. Differences with adjusted *p* value < 0.05 were considered statistically significant.

## 3. Results

### 3.1. Fluoride Concentration of Commercially Available Toothpastes

**Figure 2:**
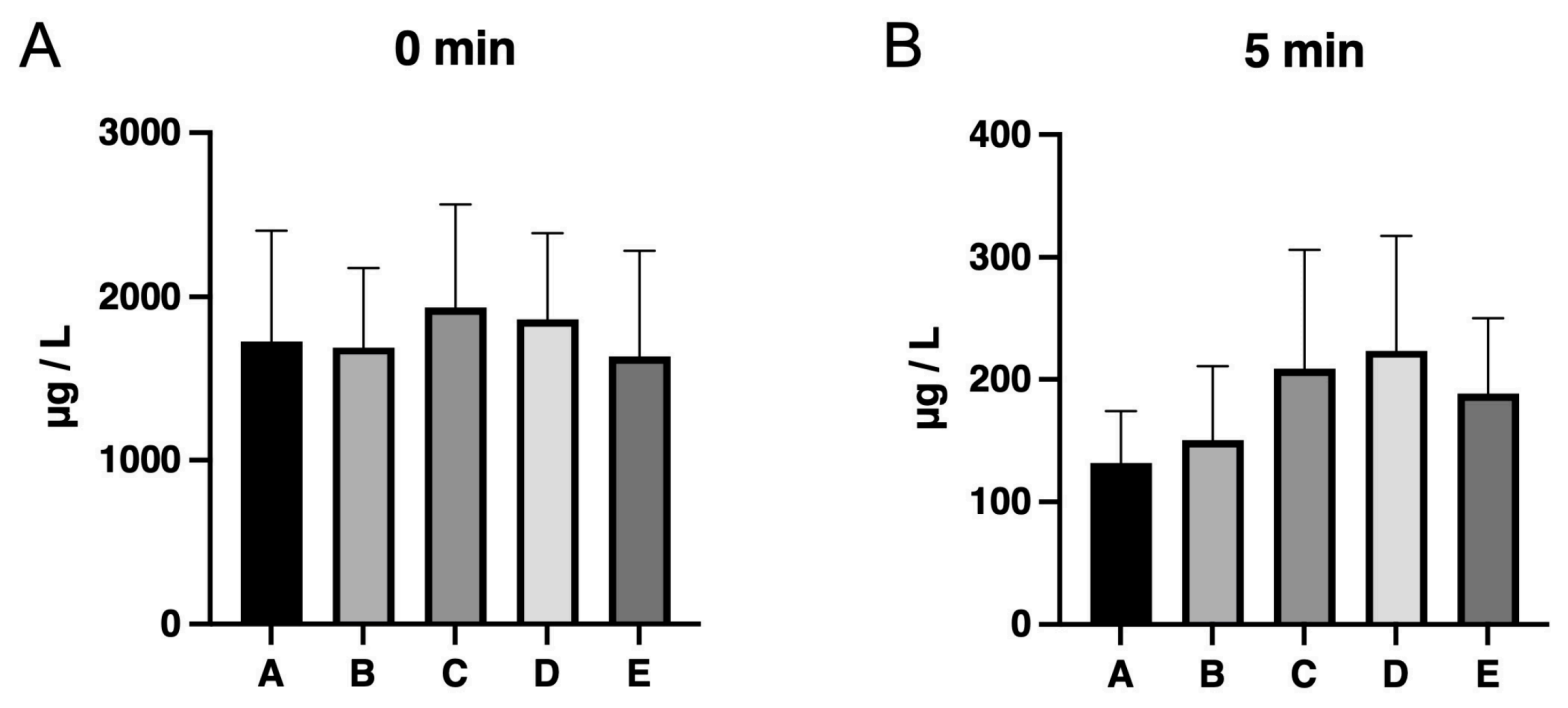

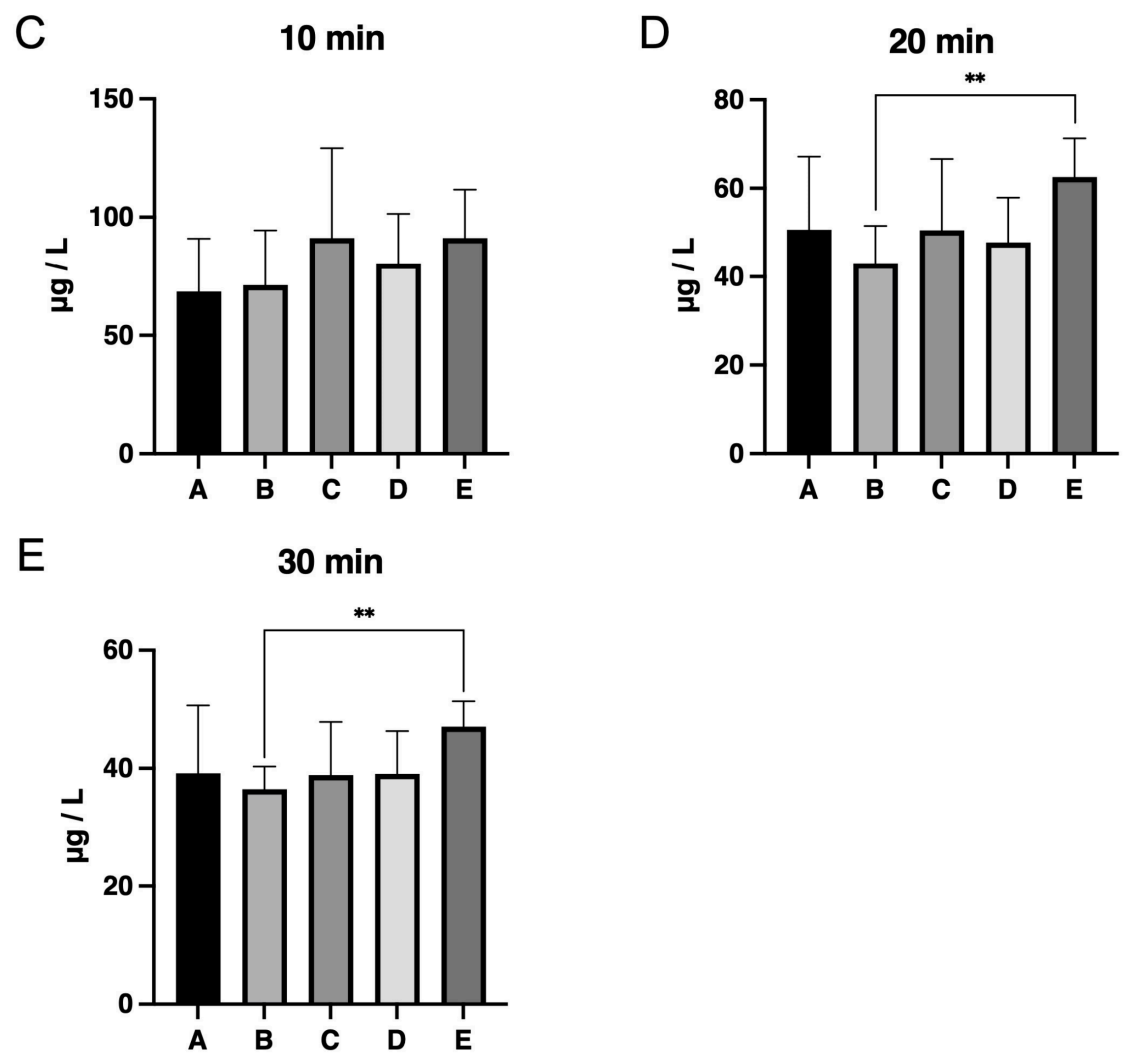
Fluoride concentration of commercially available toothpastes. (**A**) 0 min, (**B**) 5 min, (**C**) 10 min, (**D**) 20 min, (**E**) 30 min. Significant differences: ** *p* < 0.01.

Although there were no differences among the five types of toothpaste at 0 min, the fluoride ion concentration of toothpaste D was the highest after 5 min (Figure 2A,B). Subsequently, toothpaste E showed the highest fluoride concentration at all time points, and was significantly higher than toothpaste B at 20 and 30 min (*p* < 0.01) (Figure 2C–E). Among the toothpastes with abrasives, toothpaste C recorded higher fluoride concentrations at 0, 5, and 10 min; however, no differences were observed thereafter.

### 3.2. Fluoride Concentration of Toothpastes with or Without Abrasive Components

**Figure 3:**
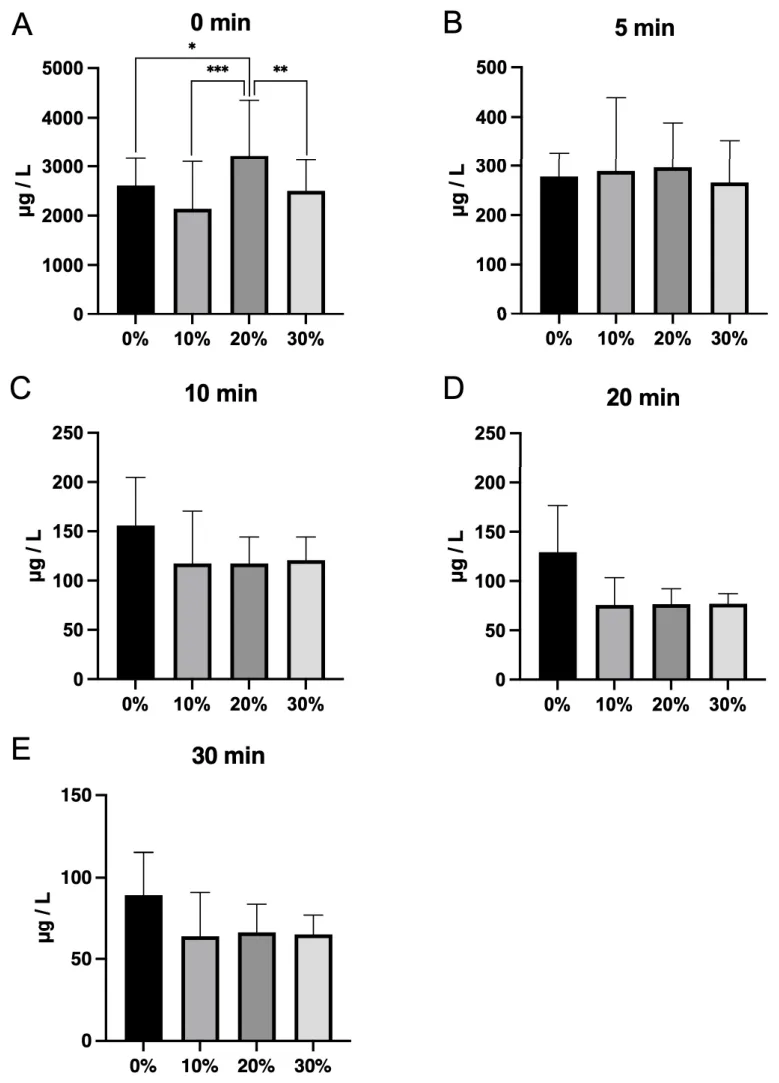
Fluoride concentration of toothpastes with different amounts of abrasive ingredients. (**A**) 0 min, (**B**) 5 min, (**C**) 10 min, (**D**) 20 min, (**E**) 30 min. Significant differences: * *p* < 0.05, ** *p* < 0.01, and *** *p* < 0.001.

Prior to the experiment, the fluoride ion concentrations of 0.5 g of each toothpaste with 0%, 10%, 20%, and 30% abrasive components were measured, and no significant differences were recorded (Supplemental Figure S1). At 0 min, the 20% toothpaste showed the highest fluoride concentration, with a significant difference compared with the other toothpastes (*p* < 0.05), but no significant difference was observed at 5 min (Figure 3A,B). After 10 min, the fluoride concentration of the 0% toothpaste began to increase, and at 20 and 30 min, it remained the highest among all the toothpastes (Figure 3C–E).

### 3.3. Fluoride Concentration of Apatite Pellets After Toothbrushing

**Figure 4:**
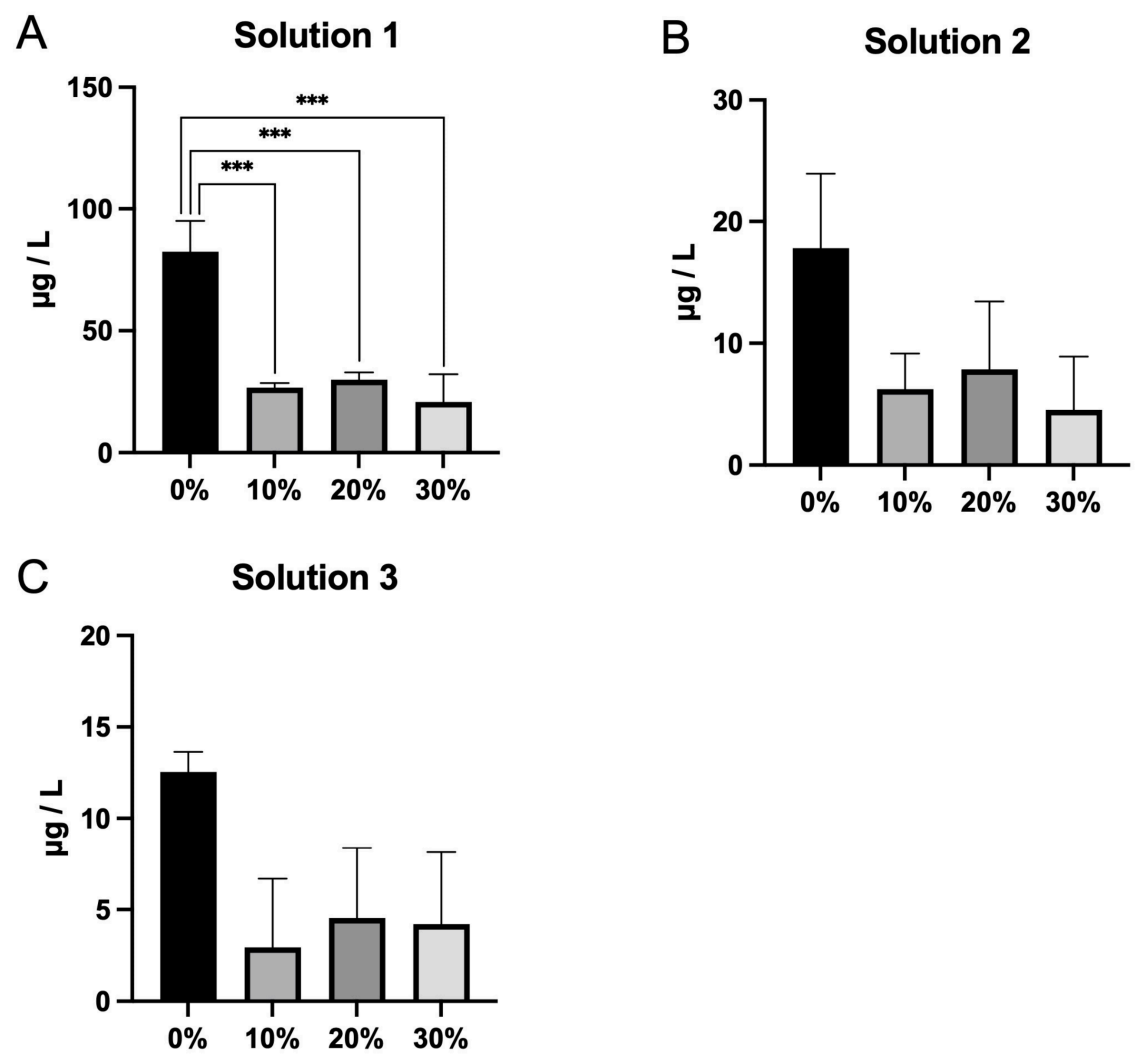
Fluoride concentration of apatite pellets after brushing with different amounts of abrasive ingredients. (**A**) Solution 1, (**B**) Solution 2, (**C**) Solution 3. Significant differences: *** *p* < 0.001.

First, we brushed the pellets without toothpaste and measured the fluoride ion concentrations for solutions 1 through 3, and confirmed no change in any of the three solutions and no fluoride ions were released from the apatite pellets (Supplemental Figure S2). We then compared the solutions of pellets brushed with toothpaste containing different amounts of abrasive components. In solution 1, the 0% toothpaste recorded significantly higher fluoride concentrations than the other three toothpastes (*p* < 0.001) (Figure 4A). Even after multiple rinsing steps, the fluoride concentration of the 0% toothpaste was consistently higher than the other toothpastes (Figure 4B,C).

## 4. Discussion

Toothpastes often contain a range of ingredients; however, few studies have focused on the interactions between these components. In this study, we investigated the effect of abrasive ingredients on fluoride concentration in the oral cavity. When the fluoride ion concentration rates of five types of toothpaste containing 1450 ppm of fluoride were measured at 5, 10, 20, and 30 min, the abrasive-free toothpastes consistently recorded the highest values. These results suggest that the presence or absence of abrasive ingredients may influence fluoride concentration. Of the three toothpastes with abrasive ingredients, toothpaste C, which contained hydrated silica, recorded a higher fluoride concentration at 0, 5, and 10 min when compared with toothpastes A and B, which contained silica. Silica abrasives are gentle on the teeth and gums and are highly effective at removing plaque, while the properties of hydrated silica vary depending on factors such as moisture content, particle shape, and particle size distribution [12]. Further research is needed to determine which components of hydrated silica influence the concentration of fluoride.

Based on the results obtained with the commercially available products, we decided to adjust the concentration of abrasive ingredients in the same toothpaste and conduct another comparison. At 0 min, the 20% toothpaste showed a significantly higher fluoride concentration than the other toothpastes; however, no significant difference was observed at 5 min. This may be because the 0 min measurement was taken immediately after brushing, and variability due to rinsing may have been reflected in the results. At 10 min, the fluoride ion concentration in the 0% toothpaste began to exceed that of the other toothpastes, and was the highest at 20 min among four toothpastes. However, the magnitude of this difference had decreased by 30 min, possibly because the effects of the toothpaste had already worn off, allowing the oral environment to return to its original state. As described above, the anticaries effect of fluoride is primarily attributed to the sustained presence of low concentrations of fluoride in the oral environment [9]. However, it remains unclear to what extent the differences in fluoride concentration over time observed in the present study translate into clinically meaningful anticaries effects. Although the abrasive-free toothpaste maintained higher fluoride concentrations for a longer period than the other toothpastes, the clinical significance of this finding remains to be determined in future studies.

We also investigated the concentration of fluoride using hydroxyapatite pellets, which are sintered from hydroxyapatite, a component of teeth. The pellicle, as a biological membrane, has been suggested to contribute to the formation and retention of protective precipitates through modulation of fluoride adsorption, diffusion, and desorption [13]. The pellets used in the present study had smooth surfaces and lacked factors that may influence fluoride retention in the oral cavity, such as the acquired pellicle. However, pellets brushed with a toothpaste containing no abrasive ingredients continued to release high levels of fluoride ions into the washing solution even after multiple washings. These results suggest that abrasive ingredients play a significant role in fluoride concentration and may adhere to and remain on the surface of the apatite pellets. Future studies incorporating factors that influence fluoride retention in the oral cavity, such as the pellicle, may allow for a more clinically relevant evaluation that better reflects the in vivo oral environment. In studies conducted on humans, we evaluated mouthwash containing saliva, and in studies using pellets, we investigated the concentration of fluoride released from the pellets. Baez-Polan et al. (2025) reported that the use of a calcium treatment before a fluoride rinse can increase intraoral fluoride concentration and result in sustained levels of fluoride in saliva and the dental biofilm [14]. Although the present study examines the concentration of fluoride in saliva and teeth, additional research is necessary to investigate changes in the dental biofilm and soft tissues such as the gums.

This study had some limitations. First, this study was an exploratory investigation, and it had a very small sample size. Additional studies with a larger sample size are needed in the future. Second, in the in vitro apatite pellet experiments, this study could not distinguish between fluoride bound to hydroxyapatite and residual dentifrice adhering to the surface. Although we also concluded that fluoride concentration was affected by the presence or absence of abrasive components, the underlying cause of these effects remained unclear. As the concentration of abrasive particles increased, the viscosity decreased, and this change in the toothpaste’s physical properties may have had an impact. However, even at a concentration of only 10% of abrasive, the fluoride ions released from hydroxyapatite pellets decreased significantly. Further studies are needed to clarify the underlying mechanism, taking into account the possibility that factors other than viscosity are involved. In addition, this study used only silica as the abrasive ingredient. There are many kinds of abrasive agents in the dental field with improvements being made every day [12]. It remains to be determined whether similar effects would be observed with other abrasive agents, such as calcium carbonate or alumina, and these alternatives warrant further research.

## 5. Conclusions

Even a 10% concentration of abrasive in toothpastes may affect the concentration of fluoride in the oral cavity. Furthermore, significant differences were observed among apatite pellets, suggesting that fluoride remains in the oral cavity for a longer period by adhering to hard tissues such as teeth when toothpastes without abrasive ingredients are used.

## Figures and Tables

**Table 1:** Characteristics of each toothpaste.

Toothpaste	Fluoride Concentration	Abrasive Ingredients	Foaming Agent	Binder	Flavoring Agent
Toothpaste A	1450 ppm	Silica	Cocamidopropyl Betaine, Sodium Lauryl Sulfate	Sodium Polyacrylate	Flavor, Xylitol, Sodium Saccharin
Toothpaste B	1450 ppm	Silica	Cocamidopropyl Betaine, PEG Hydrogenated Castor Oil, Polyoxyethylene Stearyl Ethers, Sodium Lauryl Sulfate	Xanthan Gum	Flavor, Xylitol
Toothpaste C	1450 ppm	Hydrated silica	Cocamidopropyl Betaine	Hydrated Silica	Flavor, Sodium Saccharin, Sucralose
Toothpaste D	1450 ppm	None	None	Xanthan Gum, Carrageenan, Polyvinylpyrrolidone	Flavor, Xylitol
Toothpaste E	1450 ppm	None	Cocamidopropyl Betaine	Sodium Carboxymethyl Cellulose	Flavor, Sodium Saccharin

## Data Availability

The data are available from the corresponding author upon reason-able request.

## Supplementary Materials

The following supporting information can be downloaded at https://www.mdpi.com/article/10.3390/dj14090584/s1. Supplemental Figure S1. Amount of fluoride contained in toothpaste; Supplemental Figure S2. Fluoride concentration of apatite pellets after brushing without toothpaste.
